# Evaluating Deep Learning-Based Commercial Software for Detecting Ischemic Lesions on DWI in Stroke Patients

**DOI:** 10.3390/diagnostics15182357

**Published:** 2025-09-17

**Authors:** Ceren Alis, Elvin Ay, Gencer Genc, Serpil Bulut

**Affiliations:** 1 Department of Neurology, Sisli Hamidiye Etfal Training and Research Hospital, 34396 Istanbul, Turkey; gencer.genc@sbu.edu.tr (G.G.); serpil.bulut@sbu.edu.tr (S.B.); 2 Department of Neurology, School of Medicine, Acibadem Mehmet Ali Aydinlar University, 34752 Istanbul, Turkey; elvin.ay@acibadem.edu.tr

**Keywords:** ischemic stroke, diffusion-weighted imaging, magnetic resonance imaging, deep learning, artificial intelligence, lesion detection

## Abstract

**Background:** Recent advancements in deep learning have enabled the development of automated software to assist in ischemic lesion detection on diffusion-weighted imaging (DWI), but their real-world performance remains underexplored. This study evaluated the diagnostic performance of a commercially available, CE-marked (MDR class IIa) artificial intelligence (AI) software version 1.0 for detecting ischemic lesions on DWI and examined its sensitivity in relation to lesion-specific characteristics. **Methods:** A retrospective cohort of 235 patients with confirmed ischemic stroke who underwent DWI was analyzed. The CE-marked software’s performance was assessed at both lesion and patient-level, using expert neurologist interpretations as the reference standard. Lesion characteristics, including maximum axial size, apparent diffusion coefficient (ADC) values, slice coverage, and anatomical location, were analyzed. **Results:** The software achieved a lesion-level sensitivity of 83.51% (95% CI, 79.8–86.8%) and a patient-level sensitivity of 95.31% (95% CI, 91.8–97.6%). Undetected lesions were significantly smaller, covered fewer slices, and had higher ADC values. No significant differences were observed in detection rates by anatomical locations, vascular territories, or time from symptom onset. **Conclusions:** While the AI software demonstrated a strong patient-level sensitivity overall, it showed limitations in identifying smaller, less conspicuous lesions. These findings underscore the need to optimize deep learning algorithms for better sensitivity and highlight the importance of clinician awareness regarding AI limitations in acute stroke care.

## 1. Introduction

Ischemic stroke is a profound global health challenge and remains one of the leading causes of mortality and long-term disability [1,2]. Advanced neuroimaging techniques, including computed tomography (CT) and magnetic resonance imaging (MRI), are integral to the diagnosis and management of ischemic stroke [3]. While CT is frequently preferred for its rapid imaging capabilities, which facilitate timely initiation of treatment, MRI—particularly diffusion-weighted imaging (DWI)—offers unparalleled contrast resolution, enabling precise delineation of ischemic lesions, particularly in the early stages of stroke [4,5,6].

Deep learning (DL), a specialized subset of machine learning, employs intricate neural networks to extract salient features and perform predictive tasks simultaneously [7]. Within the domain of ischemic stroke, DL methodologies, particularly convolutional neural networks (CNNs), have demonstrated significant promise in delineating ischemic cores on DWI, frequently outperforming traditional machine learning and threshold-based methods [8,9,10].

The accurate and early detection of ischemic lesions is paramount for patient triage and expediting neurologist intervention [11]. Several artificial intelligence (AI) solutions are currently available in the market for stroke diagnostics, with most earlier studies focusing on their segmentation performance, such as delineating ischemic cores [12,13]. However, there is a paucity of research evaluating the performance of commercially available AI-based software for ischemic lesion detection (i.e., triage) on DWI.

Our hospital has been utilizing a CE-marked (MDR class IIa) AI-based triage system on DWI, integrated with a mobile application, for the past year. During this time, it has facilitated timely lesion detection and improved workflow efficiency in stroke management. This long-term implementation provided the impetus to assess its diagnostic performance, which informed the selection of this software for detailed evaluation.

The objectives of this study are twofold: first, to evaluate the diagnostic accuracy of the AI-based commercially available software in detecting ischemic lesions; second, to assess its sensitivity concerning lesion-specific characteristics, with the aim of better understanding its utility, flaws, and strengths as a triage system for optimizing stroke care delivery.

## 2. Materials and Methods

### 2.1. Study Population

This was a retrospective single-center study conducted at Sisli Hamidiye Etfal Training and Research Hospital, including adult patients who were consulted by the neurology team in the emergency department for suspected stroke between August 2024 and January 2025. All patients underwent DWI as part of their diagnostic workup and were confirmed to have ischemic stroke based on clinical evaluation and imaging findings. All patients were imaged using Siemens Avanto 1.5T MRI units.

Patients included in the study were selected based on the availability of confirmed ischemic lesions on DWI. Demographic data, such as age, sex, and relevant clinical history, were collected to facilitate subgroup analyses and interpretation of the model’s performance. The ethics committee of Sisli Hamidiye Etfal Training and Research Hospital approved the retrospective review of patient records and waived the need for informed consent (Ethical approval date and number: 11/02/2025—4738).

**Figure 1:**
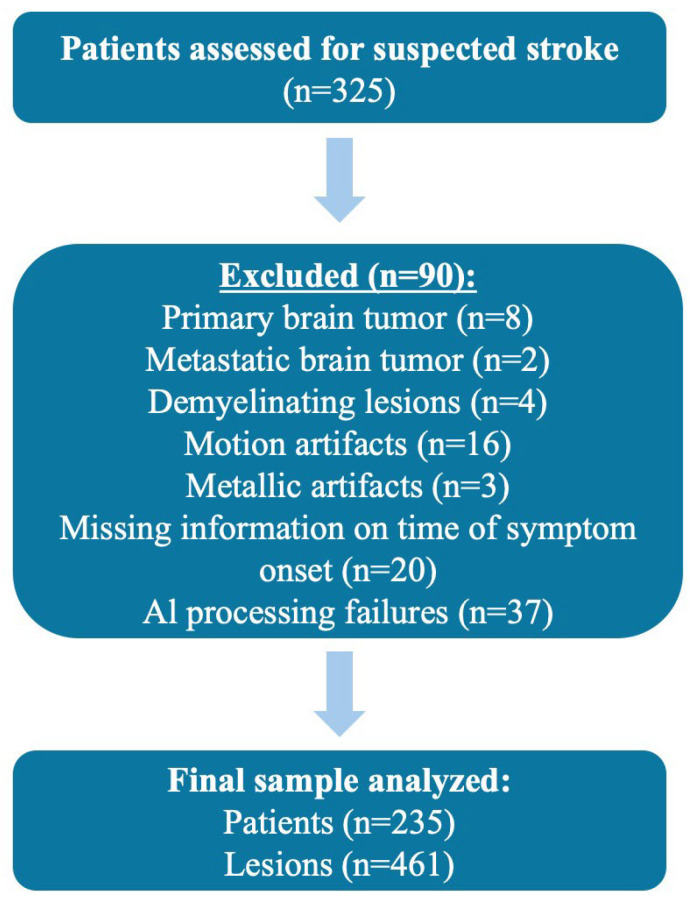
Flowchart of the study.

Patients were included in the study if they were aged 18 years or older, had a confirmed ischemic stroke based on clinical criteria and DWI findings, and had complete imaging data. Patients were excluded if they had primary brain tumors, metastatic brain tumors, or demyelinating lesions; if their DWI scans contained severe motion artifacts or metallic artifacts that hindered image interpretation; if their imaging or clinical data were incomplete, such as missing information on time of symptom onset or unavailable apparent diffusion coefficient (ADC) maps; or if the AI software failed to process the data due to known or unknown technical errors, including device malfunctions or non-responsive Picture Archiving and Communication System (PACS). Figure 1 shows the flowchart of the study.

### 2.2. Imaging Equipment

The imaging data for this study were acquired using Siemens Avanto 1.5T MRI units. The field strength for all exams was set at 1.5 Tesla. Slice thickness was maintained at 5 mm, with a field of view (FOV) of 240 mm × 240 mm. The matrix size was set to 128 × 128, and diffusion gradients were applied with two b-values, typically 0 and 1000 s/mm^2^.

### 2.3. AI Software

The AI software (hStroke_Suite DWI module V1, Hevi AI, Istanbul, Turkey) used in this work is a DL-based triage system designed to detect ischemic lesions on DWI. This module leverages a modified U-net architecture optimized for stroke detection, integrating advanced features to address the unique challenges of DWI analysis.

The underlying architecture of the AI software is based on a residual convolutional long short-term memory (ConvLSTM) U-net, which combines the strengths of CNNs and recurrent neural networks (RNNs). This hybrid architecture captures both spatial and sequential information essential for ischemic lesion detection. The encoder component extracts representative spatial features, while the decoder performs up-sampling to restore spatial resolution, ensuring precise segmentation. Skip connections between the encoder and decoder preserve spatial information, enhancing the model’s ability to localize ischemic lesions.

Unlike traditional U-net implementations, the inclusion of residual connections and ConvLSTM units allows retaining contextual information across multiple slices. This design choice overcomes the limitations of 2D networks, which lack sequential interpretability, and 3D U-net models, which require high memory capacity and may lead to loss of contextual information due to spatial down-sampling or patch-based approaches.

The model was trained on a diverse dataset of anonymized DWI scans from multiple institutions, ensuring robust performance across various scanner types and imaging protocols. The dataset included cases with ischemic lesions of varying sizes and anatomical locations, as well as normal scans. Rigorous cross-validation was conducted to ensure generalizability and external validation was performed to assess its performance on unseen data. Further information about the model can be found in an earlier work [12].

### 2.4. Integration with Clinical Workflow

The AI software integrates into the existing clinical workflow: (1) Connection to MRI units: The module is configured to receive data directly from MRI units, (2) Automated Data Transfer: Following DWI acquisition, the MRI unit sends the imaging data to the AI module through pre-configured protocols; (3) Data Processing: The module processes the data, analyzing DWI with two b-values and calculated ADC maps to detect ischemic lesions; (4) Result Delivery: Processed results are sent to the hospital’s PACS and a mobile application. For normal and ischemic cases, alerts are generated on the mobile application, facilitating timely intervention by neurologists.

### 2.5. Analysis of Patient and Lesion Characteristics

Lesion characteristics were evaluated by a board-certified neurologist with 10 years of experience in acute stroke imaging (C.A.). The reference standard was established based on the visual interpretation of DWI scans, where ischemic lesions were defined as hyperintense regions on DWI with corresponding hypointensity on ADC maps. Measurements were conducted on a dedicated workstation using specialized image-viewing software to ensure precision and consistency. The parameters measured in this study included the following:

1. Maximum Axial Size: The largest diameter of each lesion was recorded in the axial plane.
2. ADC Values: ADC values were documented to quantify the degree of diffusion restriction for each lesion.
3. Number of Slices Covered: The number of axial slices containing the lesion was noted.
4. Anatomical Location: Lesion locations were categorized by brain lobe (e.g., frontal, parietal) and vascular territories (e.g., anterior vs. posterior circulation).

Patient-related factors were assessed to evaluate their potential impact on the performance of the AI software in detecting ischemic lesions. The recorded parameters included the following:

1. Age: The age of each patient was documented to analyze potential variations in detection accuracy across different age groups.
2. Sex: Sex was recorded to evaluate any differences in detection rates between male and female patients.
3. Time from Onset to Imaging: The interval between symptom onset and imaging acquisition was recorded to determine its effect on lesion detectability.

### 2.6. Analysis of True Positive (TP) and False Negative (FN) Lesions

The analysis of true positive (TP) and false negative (FN) lesions was performed by comparing the lesions detected by the AI software with those identified by an expert neurologist, serving as the reference standard. Lesions were categorized into two groups: AI-detected lesions and undetected lesions. This grouping facilitated a detailed comparison of lesion characteristics, such as size, ADC values, and anatomical location, to identify factors that may influence the model’s performance. By evaluating these categories, the study aimed to assess the strengths and limitations of the AI software version 1.0 in ischemic lesion detection, providing insights for further optimization.

### 2.7. Statistical Analysis

The statistical analyses were performed using Python (version 3.10), employing the SciPy library for statistical computations. All data processing and analysis workflows were carried out in Python, utilizing Pandas for data manipulation and Matplotlib version 3.9 for data visualization. Continuous variables, such as maximum axial size, ADC value, slices covered by the lesion, and time from onset to imaging, were reported as means ± standard deviations (SD) alongside their minimum and maximum values. Lesion- and patient-level 95% CIs calculated using the Wilson method. Group comparisons for continuous variables were conducted using independent two-sample *t*-tests, with Welch’s correction applied where assumptions of equal variances were not met. A significance level of *p* < 0.05 was used to determine statistical significance.

Categorical variables, including anatomical locations and vascular territories, were analyzed using chi-square test. Anatomical locations were grouped into supratentorial (cerebral hemispheres and diencephalon) and infratentorial (cerebellum, brainstem) regions, while vascular territories were grouped into anterior (Middle Cerebral Artery, Anterior Cerebral Artery, Internal Carotid Artery) and posterior (Posterior Cerebral Artery, Basilar Perforators, Posterior Inferior Cerebellar Artery, Superior Cerebellar Artery) circulations to simplify comparisons. Chi-square test was applied to assess differences in distribution between AI-detected and undetected lesions for these categorical groupings.

## 3. Results

This study analyzed a cohort of 235 patients diagnosed with ischemic stroke, 57% of whom were male. The mean age of the cohort was 67.96 ± 14.05 years, with ages ranging from 22 to 100 years.

The sensitivity of the AI model was analyzed at both the lesion-level and the patient-level. At the lesion-level, the model successfully identified 385 TP lesions out of 461 total lesions, while 76 lesions were classified as FN. This corresponds to a lesion-level sensitivity of 83.51% (95% CI, 79.8–86.8%). At the patient-level, the model correctly detected ischemic stroke lesions in 224 patients, while 11 patients had no lesions identified by the model. With a total of 235 patients in the cohort, the patient-level sensitivity was calculated as 95.31% (95% CI, 91.8–97.6%).

Lesion characteristics exhibited considerable variability. The mean maximum axial size of lesions was 17.28 ± 21.11 mm, with sizes ranging from 2.8 to 126.0 mm. The mean ADC value was 557.50 ± 156.71 mm^2^/s × 10^−6^, with values spanning from 101 mm^2^/s × 10^−6^ to 1472 mm^2^/s × 10^−6^. Lesions extended across an average of 2.67 ± 2.54 slices, with a range of 1 to 17 slices. The mean time from symptom onset to imaging was 456.78 ± 320.45 min, with times ranging from 30 to 1500 min.

**Figure 2:**
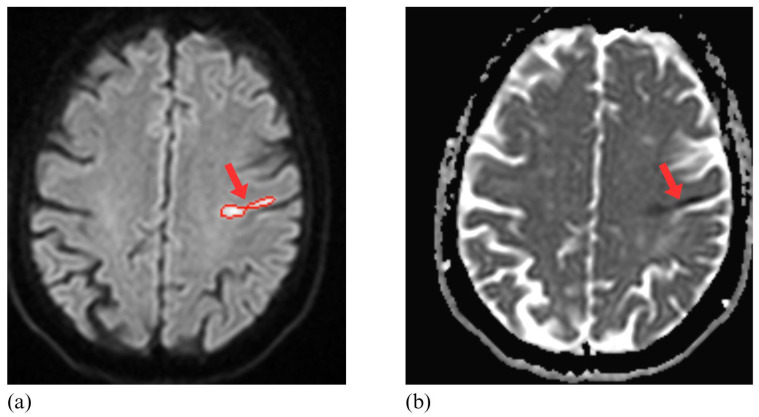
Detection of an Ischemic Lesion by Artificial Intelligence on DWI and ADC Maps. A 67-year-old woman presented with sudden-onset dysarthria and right upper limb weakness. The diffusion-weighted imaging (DWI) scan (**a**) and the apparent diffusion coefficient (ADC) map (**b**) correctly identified the ischemic lesion in the precentral gyrus (arrows) using the artificial intelligence (AI) software. On DWI (**a**), the AI delineation of the ischemic lesion is highlighted with red, indicating the automatically segmented area. The AI accurately delineated the ischemic area and generated an alert via the mobile application.

**Figure 3:**
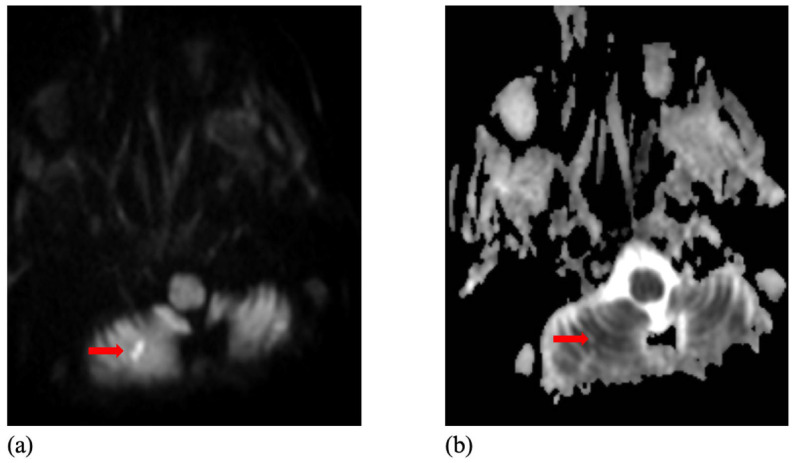
A Missed Ischemic Lesion by Artificial Intelligence on DWI and ADC Maps. A 92-year-old woman presented with acute-onset nausea, vomiting, and ataxia. The diffusion-weighted imaging (DWI) scan (**a**) and the apparent diffu-sion coefficient (ADC) map (**b**) showed an ischemic lesion in the right inferior cerebellum (arrows), which was not detected by the AI software.

Table 1 provides a detailed summary of demographic and radiological characteristics of the study sample. Figure 2 and Figure 3 illustrate representative examples of AI-detected and undetected lesions, highlighting the model’s performance across varying lesion characteristics.

### 3.1. Lesion Diameter, Slice Coverage, and ADC Values of AI-Detected Lesions and Undetected Lesions

Group comparisons between AI-detected lesions and undetected lesions revealed significant differences. Lesions identified by AI exhibited larger maximum axial sizes, averaging 19.16 ± 22.38 mm, compared to undetected lesions, which measured 7.71 ± 7.64 mm (*p* < 0.0001). ADC values were lower in detected lesions (538.62 ± 144.27) compared to undetected lesions (653.16 ± 181.54, *p* < 0.0001). Additionally, detected lesions extended across more slices on average (2.93 ± 2.67 slices) than undetected lesions (1.33 ± 0.93 slices, *p* < 0.0001).

When stratified by lesion size (Table 2), detection increased sharply with size: 48.1% (95% CI, 37.6–58.9) for <5 mm (39/81), 87.3% (81.4–91.6) for 5–10 mm (145/166), 92.7% (86.3–96.3) for 10–20 mm (102/110), and 95.2% (89.2–97.9) for ≥20 mm (99/104). Thus, nearly all false negatives clustered among the smallest lesions, whereas lesions ≥10 mm were detected with >90% sensitivity.

### 3.2. Onset-to-Imaging Time of AI-Detected Lesions and Undetected Lesions

No significant difference was observed in the mean from symptom onset-to-imaging time between the two groups. Detected lesions had a mean imaging time of 430.12 ± 300.67 min, whereas undetected lesions averaged 610.34 ± 380.21 min (*p* = 0.2053).

### 3.3. Vascular and Anatomical Distribution of AI-Detected Lesions and Undetected Lesions

When grouped anatomically, 83.5% of lesions (385/461) were supratentorial and 16.5% (76/461) were infratentorial. Detection was 83.4% (321/385) in supratentorial and 84.2% (64/76) in infratentorial lesions (*p* = 0.317).

Similarly, when vascular territories were grouped, 73.32% of lesions (338/461) were in the anterior circulation, and 26.68% (123/461) were in the posterior circulation. Among AI-detected lesions, 283 were in the anterior circulation and 102 were in the posterior circulation, while among undetected lesions, 55 were in the anterior circulation and 21 were in the posterior circulation. Chi-square test for the vascular territories also revealed no significant difference in distribution between the two groups (*p* = 0.887).

## 4. Discussion

In this real-world sample, the CE-marked AI software achieved lesion-level sensitivity of 83.51% (385/461; 95% CI, 79.8–86.8%) and patient-level sensitivity of 95.31% (224/235; 95% CI, 91.8–97.6%). Notably, lesions undetected by AI had a smaller size, covered fewer slices, and higher ADC values compared with AI-detected lesions. No significant differences were observed in detection rates based on lesion location, vascular territory, or time from symptom onset.

The discrepancy between patient-level and lesion-level sensitivity difference reflects strong triage performance—detection of at least one lesion in nearly all patients—despite missed micro-foci that were smaller, spanned fewer slices, and had higher ADC values. AI outputs were integrated as decision support (CE-marked; MDR class IIa), while final treatment decisions were made by physicians and double-checked per protocol; accordingly, missed micro-lesions did not affect acute reperfusion eligibility in this sample. Nonetheless, subtle ischemic changes may inform risk stratification and secondary prevention, underscoring AI’s complementary role rather than replacement of clinician review.

Most studies on AI-based ischemic lesion evaluation on DWI have primarily emphasized their applicability and effectiveness in segmenting lesions. However, there is limited research on the ability of DL models to detect ischemic lesions, a crucial function for timely clinical interventions.

Federau et al. trained a CNN on a dataset of approximately 3000 individuals to identify acute ischemic lesions, achieving a sensitivity of 91% and a specificity of 75% when tested on 192 individuals [14]. Federau et al. attributed their higher detection rates to the enhancement of DWI through synthetic data augmentation, which likely improved the visibility of smaller and less conspicuous lesions [14].

Similarly, Nael et al. developed a multi-contrast, 3D CNN trained on more than 13,000 clinical brain MRI studies (including 9845 for training) and evaluated its performance for acute infarction detection on external datasets. The model achieved excellent accuracy with an AUC of 0.97, sensitivity of 90%, and specificity of 97% [15]. Importantly, they demonstrated a clear size–sensitivity gradient, reporting sensitivities of 84% for lesions < 1 mL, 79% for lesions < 0.5 mL, and 72% for lesions < 0.25 mL. This gradient directly parallels our finding that missed lesions in our cohort were characteristically smaller, covered fewer slices, and exhibited higher ADC values, underscoring the universal challenge of detecting subtle ischemic foci across different AI software.

Bridge et al. trained a DWI + ADC model using 6657 classification studies and 377 segmented cases, and tested it on a 792-patient internal dataset, achieving a sensitivity of 98.4% [16]. In real-world stroke-code cohorts, sensitivity was somewhat lower (89.3% at the training site and 96.1% at the non-training site), but overall case-level discrimination remained high (AUROC 0.96–0.98). Most false negatives corresponded to sub-milliliter infarcts, and across all missed cases lesion volumes were significantly smaller than those in true positives (12.3 vs. 26.4 mL). These observations closely mirror our results, reinforcing that lesion conspicuity, rather than model generalizability, is the main determinant of detection failure.

Krag et al. externally validated a CE-marked AI software (Apollo v2.1.1, Cerebriu, København, Denmark) in 995 patients from a non-comprehensive stroke center. Compared with expert neuroradiology reads, the software achieved a case-level sensitivity of 89% and specificity of 90% [17]. Sensitivity strongly scaled with lesion volume, from 79% for lesions < 0.5 cm^3^ to 95% for 0.5–5 cm^3^ and 100% for those >5 cm^3^. These results are consistent with our lesion-level analysis, where smaller, less conspicuous lesions were more frequently missed. In contrast, our patient-level sensitivity (95.3%) was higher, reflecting that the detection of at least one lesion per patient is usually sufficient for triage.

Krag et al. observed that lesion detectability was lower for non-acute ischemic lesions (e.g., subacute and chronic), while our analysis found no significant variation in detectability based on the time from symptom onset [17]. This finding may be influenced by the acute nature of our study cohort, which primarily included patients presenting with ischemia in its early stages, as opposed to Krag et al.’s broader range of lesion stages, encompassing subacute and chronic phases. These variations highlight the influence of patient and lesion characteristics on AI performance and underscore the need for further validation across diverse clinical settings.

More recently, Krag and colleagues also cautioned that excluding diagnostically uncertain lesions—often small, infratentorial, or artifact-prone—can introduce spectrum bias and lead to overestimation of diagnostic performance [18]. This further emphasizes the importance of evaluating AI software across the full spectrum of ischemic presentations, including the most subtle and challenging cases [18].

An important strength of AI-based triage software is their potential to complement human readers in terms of speed, accuracy, and reproducibility. By rapidly processing DWI scans and delivering automated alerts, the software can reduce time-to-diagnosis in busy clinical workflows [11,16]. In our deployment, the AI pipeline operated as a zero-click, DICOM-triggered workflow: upon PACS ingestion of the DWI/ADC series, studies were automatically routed to the inference node and results returned within minutes as a dedicated AI results series. Outputs were written back to PACS as standard DICOM secondary images (color overlays), allowing readers to review AI findings directly in their usual viewport without switching applications. For interpretability, the software provides slice-wise lesion overlays on DWI, enabling rapid visual verification of candidate foci and mitigating “black-box” concerns.

Moreover, while human interpretation is subject to inter-observer variability, AI software provides consistent outputs for the same imaging data, thereby enhancing reproducibility [19]. Although our results show that small and subtle lesions remain a challenge for the current software, the combination of rapid automated detection and standardized performance highlights its value as an adjunct rather than a replacement for expert assessment [17].

Furthermore, implementation of AI-based models on emerging susceptibility-based biomarkers such as quantitative susceptibility mapping and oxygen extraction fraction may provide complementary microvascular and metabolic information [20,21].

Several limitations of our study should also be acknowledged. First, imaging data were obtained exclusively from Siemens Avanto 1.5T scanners at a single comprehensive stroke center, which may limit generalizability across institutions, vendors, and field strengths and underscores the need for multi-center validations. We used the pre-specified operating threshold of the CE-marked software without site-specific tuning to reduce overfitting risk; nevertheless, hardware- and protocol-level differences could alter the detectability of subtle infarcts. In our cohort, detection was primarily associated with lesion conspicuity rather than vascular territory or posterior vs. anterior circulation, but these findings do not obviate the need for external validation.

Second, we did not assess inter-rater variability in this study. Prior reports indicate moderate–substantial inter-observer agreement for acute-stroke DWI overall (κ ≈ 0.60–0.67 among emergency physicians; κ up to ~0.63–1.00 across sequences in early presenters) [19,22], but variability increases for small/subtle lesions and posterior circulation strokes, which also exhibit higher early DWI-negative rates [23,24]. Our false-negative lesions (smaller, fewer slices, higher ADC) fall within this challenging spectrum. Thus, the AI’s lesion-level sensitivity should be interpreted alongside the known range of human reader variability, whereas its high patient-level sensitivity supports triage use. Future studies should include multi-reader designs to directly benchmark AI against human variability and must guard against spectrum bias when handling diagnostically uncertain cases [25].

Third, consistent with previous reports, the AI software in our study struggled particularly with small lesions, higher ADC values, and lesions covering fewer slices, as these characteristics reduce conspicuity and increase the likelihood of being missed by automated algorithms. Importantly, recent work has also shown that excluding diagnostically uncertain lesions—often corresponding to such small or subtle findings—can introduce spectrum bias and substantially overestimate diagnostic performance [18].

Fourth, while our focus was on lesion detection rather than false positives, AI performance in stroke mimics remains a concern, as motion artifacts, DWI quality variations, and non-ischemic abnormalities can generate erroneous outputs [26]. Although infrequent in our cohort, these false positives were not systematically quantified and warrant future prospective evaluation.

Finally, the patient cohort was predominantly composed of cases with acute ischemia, precluding a robust assessment of the AI software’s performance for subacute or chronic lesions.

## 5. Conclusions

In conclusion, while the commercially available CE-marked (MDR class IIa) software version 1.0 for detecting ischemic lesions on DWI demonstrated relatively high sensitivity on a patient-level, it still failed to detect approximately one-sixth of ischemic lesions on a lesion-level. Our findings revealed that undetected lesions were smaller in size, covered fewer slices, and exhibited higher ADC values compared to AI-detected lesions. These results underscore the importance of physicians being aware of the software’s limitations and highlight the need for developers to address these shortcomings in future iterations of the software.

## Figures and Tables

**Table 1:** Lesion-level characteristics of the study sample.

Variable	Entire Lesion Set (N = 461)	Detected by AI (N = 385)	Undetected by AI (N = 76)	*p*-Value
**Maximum Axial Size (mm)**	17.28 ± 21.11 (Min: 2.8, Max: 126.0)	19.16 ± 22.38 (Min: 3.2, Max: 126.0)	7.71 ± 7.64 (Min: 2.8, Max: 45.5)	<0.0001
**ADC Value (×10^−6^ mm^2^/s)**	557.50 ± 156.71 (Min: 101, Max: 1472)	538.62 ± 144.27 (Min: 101, Max: 1220)	653.16 ± 181.54 (Min: 235, Max: 1472)	<0.0001
**Slices Covered by Lesion**	2.67 ± 2.54 (Min: 1, Max: 17)	2.93 ± 2.67 (Min: 1, Max: 17)	1.33 ± 0.93 (Min: 1, Max: 7)	<0.0001
**Time from Onset (minutes)**	456.78 ± 320.45 (Min: 30, Max: 1500)	430.12 ± 300.67 (Min: 30, Max: 1500)	610.34 ± 380.21 (Min: 90, Max: 1400)	0.2053
**Anatomical Distribution**				
Supratentorial	385/461 (83.5%)	321/385 (83.37%)	64/76 (84.2%)	
Infratentorial	76/461 (16.5%)	64/385 (16.62%)	12/76 (15.78%)	0.317
**Vascular Distribution**				
Anterior Circulation	338/461 (73.32%)	283/385 (73.51%)	55/76 (72.37%)	
Posterior Circulation	123/461 (26.68%)	102/385 (26.49%)	21/76 (27.63%)	0.887

ADC: Apparent Diffusion Coefficient; AI: Artificial intelligence; Max: Maximum; Min: Minimum; mm: Millimeter.

**Table 2:** Detection performance of the ai software stratified by lesion size.

Lesion Size	Detected/Total	Detection Rate	95% CI
<5 mm	39/81	48.1%	37.6–58.9
5–10 mm	145/166	87.3%	81.4–91.6
10–20 mm	102/110	92.7%	86.3–96.3
≥20 mm	99/104	95.2%	89.2–97.9

CI: Confidence interval; mm: Millimeter.

## Data Availability

The data that support the findings of this study are available from the corresponding author upon reasonable request, due to privacy concerns.
